# Needlestick and sharps Injuries among dental practitioners: a cross-sectional study of prevalence, risk factors, and postexposure management

**DOI:** 10.3389/fpubh.2026.1876433

**Published:** 2026-07-13

**Authors:** Basem H. AL-Huthaifi, Ehab M. Abdu, Abdullmalik N. Al-Hamati, Baker M. Abdullah, Khalid Aldhorae, Taghreed A. Al-kibsi, Mohammed A. Al-labani, Mohammed M. Al Moaleem, Emad M. Shaamala, Alia M. Alshatter, Doa'a W. Alguneed, Shahad M. Almatari, Maram N. Al-Sanfi, Fatima A. Al-Dheeb, Maram A. Al-Hajri

**Affiliations:** 1 Faculty of Dentistry, Ibn Al-Nafis University for Medical Sciences, Sana'a, Yemen; 2 Department of Oral and Maxillofacial Surgery, Faculty of Dentistry, Sana'a University, Sana'a, Yemen; 3 Faculty of Dentistry, University of Sciences and Technology, Sana'a, Yemen; 4 Orthodontic Department, Faculty of Dentistry, Thamar University, Thamar, Yemen; 5 Orthodontic Department, College of Dentistry, University of Sciences and Technology, Sana'a, Yemen; 6 Department of Prosthetic Dental Science, College of Dentistry, Jazan University, Jazan, Saudi Arabia; 7 Health Informatics Program, Department of Computer Science, Metropolitan College, Boston University, Boston, MA, United States

**Keywords:** cross-sectional study, dental students, needlestick injuries, occupational exposure, post-exposure prophylaxis, sharp injuries, Yemen

## Abstract

**Background:**

Needlestick and sharp injuries (NSIs) are a significant occupational hazard for dental practitioners, carrying a substantial risk of bloodborne pathogen transmission. This study aimed to determine the prevalence, characteristics, and factors associated with NSI exposures among dental practitioners in Sana'a, Yemen.

**Materials and methods:**

An online survey was conducted among dental practitioners at university dental hospitals, public health centers, and private clinics in Sana'a, Yemen. The self-administered questionnaire collected information on demographic background, history of NSIs, post-exposure practices, infection control compliance, and perceptions. Multivariable logistic regression was used to identify factors independently associated with lifetime NSI.

**Results:**

The lifetime prevalence of NSI was 88.18% (373/423). Among those with NSI, 36.19% reported one injury in the past 12 months, and 24.66% reported four or more injuries. The most common device was the syringe needle (61.66%), and the most frequent procedure was local anesthesia administration (39.14%). Only 11.26% of injured participants underwent post-exposure testing for bloodborne viruses, and only 9.38% received post-exposure prophylaxis. In multivariable analysis, treating more than 12 patients per day was independently associated with increased odds of NSI (adjusted odds ratio 3.50; 95% CI 1.45–8.46; *p* = 0.005). No other demographic, infection control practice, or resource variable remained significant after adjustment.

**Conclusion:**

The extremely high prevalence of NSI among dental practitioners in Sana'a, Yemen, together with very low uptake of post-exposure measures, represents a critical occupational safety failure. High patient volume emerged as a potentially modifiable associated factor. Interventions should be evaluated to prioritize workload management, universal access to safety-engineered devices, and systematic post-exposure protocols, alongside educational efforts to counter the perception that NSIs are inevitable.

## Introduction

Needlestick and sharps injuries (NSIs) are a significant occupational hazard for healthcare workers worldwide, acting as a major route for the transmission of bloodborne pathogens such as hepatitis B virus (HBV), hepatitis C virus (HCV), and human immunodeficiency virus (HIV) (1, 2). According to the World Health Organization (WHO), it is estimated that of the 35 million healthcare workers worldwide, approximately 3 million experience percutaneous exposure to bloodborne pathogens each year, with over 90% of these injuries occurring in developing countries (3).

Dental practitioners are at increased risk of sustaining needle-stick injuries (NSIs) due to the congested working conditions in the oral cavity, where visibility of sharp instruments is limited (4). The routine use of local anesthetics, rotary instruments, and sharp tools increases the likelihood of such injuries (5). Systematic reviews conducted in the past decade show that the frequency of NSIs among dental professionals is among the highest in the world (5, 6). In a meta-analysis completed in 2025, it was reported that 44% of dental assistants had sustained an NSI, primarily due to cleaning instruments and handling syringes (7).

The highest numbers of NSIs in dentistry occur among dental students and early-career dental practitioners (6–8). Rates of exposure to NSIs among dental practitioners have been shown to vary widely, with studies reporting rates ranging from 23% to 76%, with the greatest exposure occurring in final-year students due to increased clinical activity and participation in more complex dental procedures (6, 8–10). A meta-analysis by Huang et al. found that final-year dental students are significantly more likely to experience NSIs than their junior counterparts (6).

The impact of improper post-exposure treatment in healthcare environments is well-documented. According to international guidelines, essential steps include immediate wound care, timely injury reporting, assessment of the source patient, baseline and follow-up testing, and post-exposure prophylaxis (PEP) based on exposure circumstances (11). Initiating PEP promptly after exposure can reduce the risk of HIV transmission by approximately 81% (9). However, research reveals significant gaps in practice, such as a lack of knowledge regarding the source patient's pathogen status in 63.9% of cases and follow-up underreporting rates exceeding 40% (12, 13). For example, in Uganda, only 16.5% of injured students had verified the HIV/HBV status of the source patient (14).

Numerous factors contribute to noncompliance with infection control practices, categorized as individual (age, gender, experience), workplace (job responsibilities, patient volume, procedure type), and organizational factors (safety devices, infection control committees, safety culture) (4, 15, 16). The literature indicates a high prevalence of needlestick injuries (NSIs) in the Middle East and North Africa, with rates ranging from 29.8% to 84.1% (10, 13, 16–18). Common issues include frequent needle recapping, inadequate use of personal protective equipment (PPE), and insufficient post-exposure follow-up. Despite extensive research, few studies use rigorous multivariable analysis to investigate these factors across all levels of dental practitioners simultaneously. Therefore, this study aimed to estimate the lifetime and 12-month prevalence of NSIs among dental practitioners in Sana'a, Yemen, and to characterize the devices, procedures, and circumstances most frequently associated with these injuries.

## Methodology

### Study design

This cross-sectional analytical study was conducted among dental practitioners in Sana‘a, Yemen between January and February, 2026. Ethical approval was obtained from Ibn Al-Nafis University for Medical Sciences, Sana'a, Yemen (*r* = 196 on 21 December 2025). This study was performed in accordance with principles of the Helsinki Declaration (19).

### Study population and sampling

The target population comprised dental students (5th year), interns, general dentists, postgraduate dentists, and dental specialists. Participants were recruited from university dental hospitals, public health centers, and private clinics across Sana'a. Sample size was calculated using the single population proportion formula (20), with *Z* = 1.96 (95% confidence interval), *p* = 50% (to maximize sample size given previously reported prevalence variation (4, 6)), and *d* = 5% margin of error, yielding a minimum of 385 participants; however, because convenience sampling was employed, this calculation serves only as a target for recruitment and does not imply a representative sample. To account for potential non-response, the target was increased by 20% to 462.

### Instrument development, validity and pilot test

A comprehensive self-administered online survey was developed and modified from prior validated tools (6, 16, 21–24). The initial version underwent review and evaluation by a multidisciplinary panel of five experts (three consultants from oral surgery, public health and infection control). Each reviewer independently rated each item for relevance, clarity, simplicity, and ambiguity using a 4-point Likert scale (1 = not relevant, 2 = somewhat relevant, 3 = quite relevant, and 4 = highly relevant). The Content Validity Index (CVI) was calculated; items with a CVI below 0.80 were revised or eliminated. This process resulted in the removal of two items and the rewording of four items. Face validity was determined by conducting a pilot study with 30 dental practitioners who were excluded from the final analysis. The 30 dental practitioners were asked to provide feedback regarding the clarity, comprehensibility, and length of the instrument. The instrument was revised based upon their feedback, mostly by rewording unclear items and reordering questions to improve flow.

### Reliability test

The reliability of the instrument was measured by internal consistency and test-retest methods. SPSS (version 25) was used to compute Cronbach's alpha, resulting in a coefficient of 0.875, which is considered good reliability. Test-retest reliability was established by administering the instrument to a separate sample of 30 dentists twice during a 4-week interval. Intra-rater reliability was calculated using Cohen's kappa, with values ranging from 0.76 to 0.88 across items (overall κ = 0.84), which indicates substantial agreement.

### Questionnaire parts

The final instrument contained 30 questions organized into five sequential parts, each designed to systematically evaluate the prevalence, characteristics, and factors associated with needlestick and sharp injuries (NSI) among dental practitioners. The first part covered demographic and professional background. It included seven questions: gender (female or male), age (20–29, 30–39, or ≥40 years), years in clinical practice (< 3, 3–10, or >10 years), highest educational level (5th year student, intern, general dental practitioner, postgraduate, or specialist), practice type (private, governmental, academic, or mixed), average number of patients per day (≤ 12 or >12), and average work hours per day (≤ 8 or >8).

The second part focused on needlestick and sharp injury history. Seven questions asked about lifetime NSI experience, the number of NSI sustained in the past 12 months, the device involved in the most recent injury, the procedure being performed at the time, whether the device was contaminated, the body part injured, and the immediate post-exposure actions taken.

The third part related to infection control practices and consisted of seven items, each rated on a three-point Likert scale (always, sometimes, never). Practices included wearing gloves, mask, and gown; use of eye protection; high-volume suction; one-hand recapping; use of safety-engineered devices; immediate sharps disposal; and avoidance of passing sharp instruments by hand.

The fourth part concerned infection control resources at the training site and included four yes/no questions about the availability of a designated infection control unit or officer, regular NSI prevention training, an adequate supply of personal protective equipment (PPE), and safety-engineered devices.

The final part assessed participants' perceptions of NSI prevention and comprised five statements, each rated on a five-point Likert scale (from strongly agree to strongly disagree). The statements addressed the unavoidability of NSI, the importance of reporting, anxiety about blood-borne virus transmission, the benefit of more hands-on training, and the perceived institutional prioritization of student safety.

### Data collection

Data were collected between January and February 2026 using a self-administered online questionnaire hosted on a secure platform (Google Forms). A unique survey link was distributed via official institutional email lists and professional messaging groups to all eligible dental practitioners. Participation was voluntary, and electronic informed consent was obtained from each participant prior to accessing the questionnaire. A total of 435 completed the questionnaire. After excluding 12 questionnaires due to incomplete responses or missing data, 423 were included in the final analysis.

### Statistical analysis

Data were exported to a spreadsheet, cleaned and analyzed using statistical software (IBM SPSS Statistics, version 25.0 for Windows; IBM Corp., Armonk, NY, USA). Descriptive statistics were calculated as frequencies and percentages for categorical variables; lifetime NSI prevalence was estimated with its 95% confidence interval. Bivariate associations were examined using Pearson's chi-square test (or Fisher's exact test for expected cell counts < 5). Multivariable logistic regression identified independent factors associated with lifetime NSI, with all variables entered simultaneously. Results are presented as adjusted odds ratios (aOR) with 95% confidence intervals (CI). Model fit was assessed using the Hosmer-Lemeshow goodness-of-fit test, and variance explained was estimated by Nagelkerke's R^2^. *P* < 0.05 was considered significant.

## Results

### Demographic and professional characteristics

**Figure 1:**
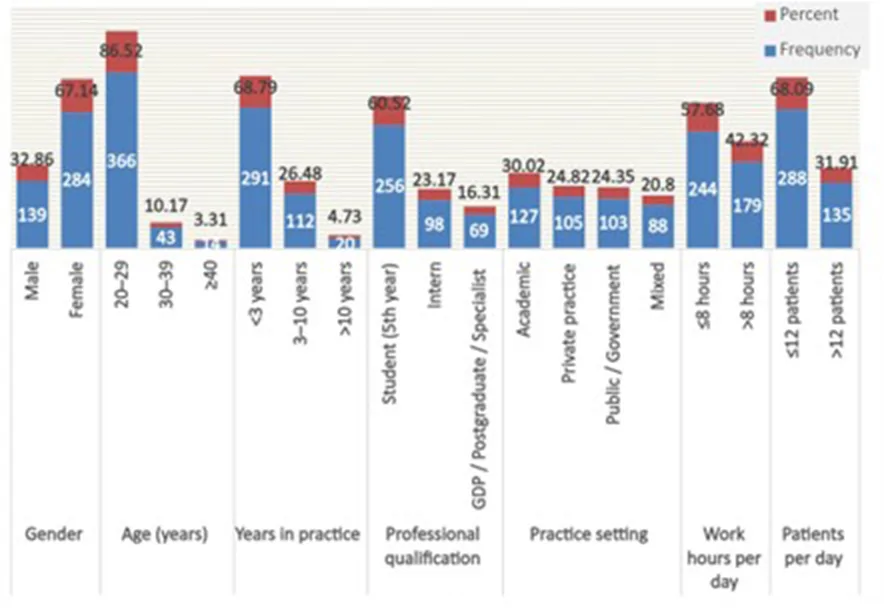
Demographic and professional characteristics of participants (*n* = 423).

A total of 423 participants completed the questionnaire. The sample was predominantly female (284, 67.14%), with most respondents aged 20–29 years (366, 86.52%) and having less than 3 years of clinical experience (291, 68.79%). Fifth-year dental students constituted the largest professional group (256, 60.52%), followed by interns (98, 23.17%). Participants represented a range of practice settings: academic institutions (30.02%), private practices (24.82%), public or government facilities (24.35%), and mixed settings (20.8%). The majority reported treating 12 or fewer patients per day (68.09%) and working 8 h or less per day (57.68%). Full demographic details are presented in Figure 1.

### Prevalence and frequency of needlestick and sharp injuries

**Figure 2:**
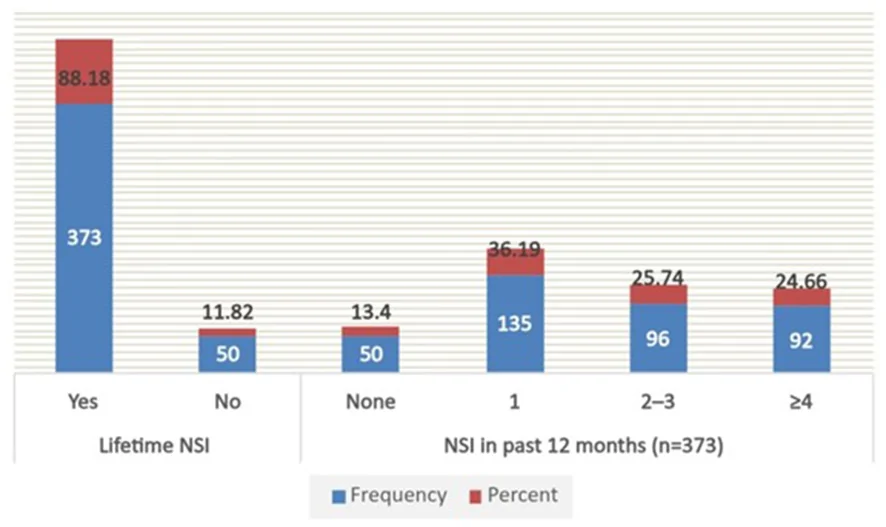
Prevalence and frequency of needlestick and sharp injuries.

Needlestick and sharp injuries were highly prevalent. Overall, 373 participants (88.18%) reported experiencing at least one such injury during their dental training. Among those with a lifetime history of NSI, 135 (36.19%) reported one injury in the preceding 12 months, 96 (25.74%) reported two to three injuries, and 92 (24.66%) reported four or more injuries. Only 50 participants (13.4%) with a lifetime NSI had no injury in the past 12 months (Figure 2).

### Characteristics of the most recent NSI

**Figure 3:**
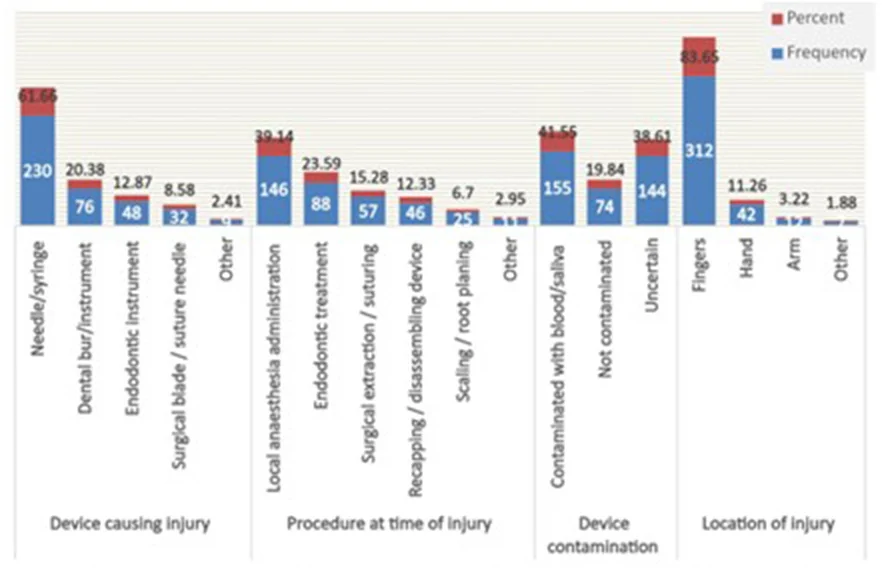
Characteristics of the most recent NSI (*n* = 373).

As shown in Figure 3. The most common device causing the most recent injury was a syringe needle (61.66%), followed by dental bur/instrument (20.38%) and endodontic instruments (12.87%). The most frequent procedures at the time of injury were local anesthesia administration (39.14%), endodontic treatment (23.59%), and surgical extraction or suturing (15.28%). In 41.55% of cases, the device was known to be contaminated with blood or saliva, while in 19.84% it was reported as uncontaminated and in 38.61% the contamination status was uncertain. The finger was the most commonly injured body part (83.65%), followed by the hand (11.26%) and arm (3.22%).

### Immediate post-exposure management practices

**Figure 4:**
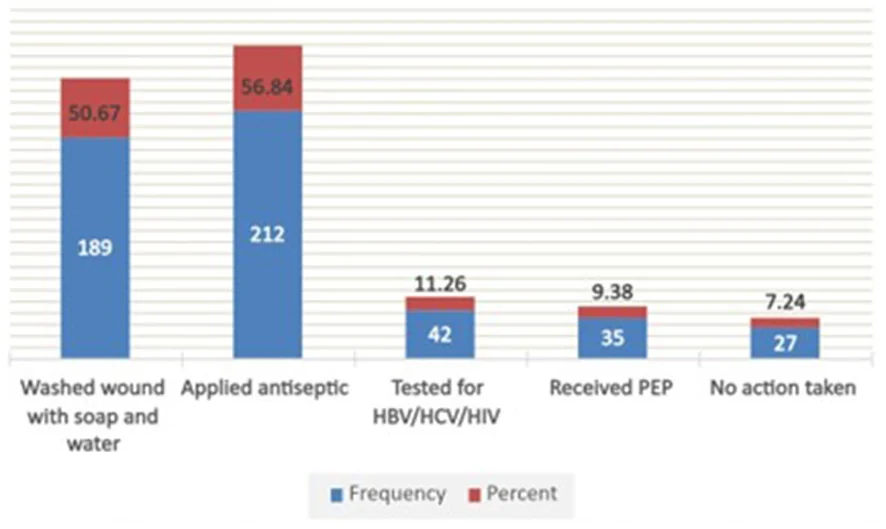
Immediate post-exposure management practices (*n* = 373).

Among participants who experienced an NSI, wound care practices were variable: 50.67% washed the wound with soap and water, and 56.84% applied an antiseptic. Critically, only 11.26% underwent post-exposure testing for bloodborne viruses (HBV, HCV, or HIV), and only 9.38% received post-exposure prophylaxis (PEP). For 7.24% of participants, no action was taken following the injury (Figure 4).

### Self-reported infection control compliance

**Figure 5:**
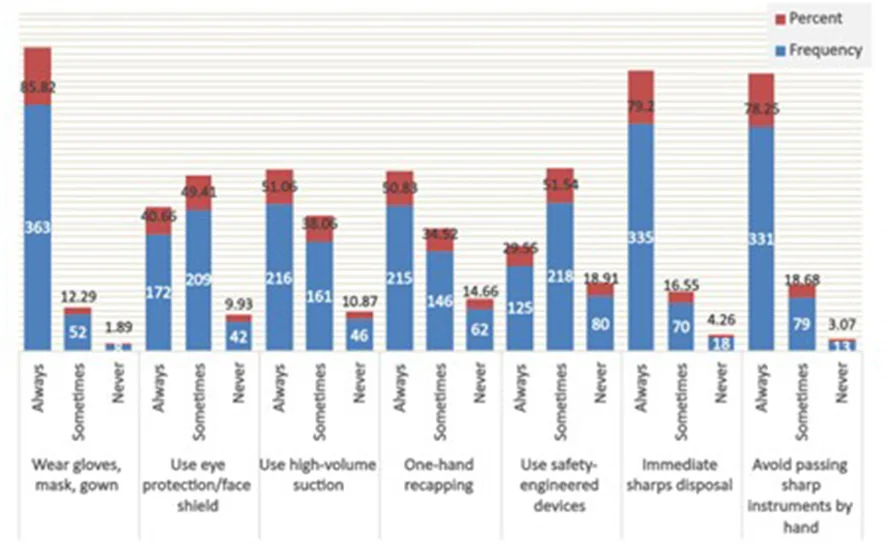
Self-reported infection control compliance (*n* = 423).

Compliance with infection control practices varied considerably. While the majority reported “always” wearing gloves, masks, and gowns during patient care (85.82%), other practices showed much lower adherence. Only 40.2% consistently used eye protection or a face shield, and 51.06% reported always using high-volume suction. One-hand recapping was always practiced by 50.83% of participants. Critically, use of safety-engineered devices was the least consistently adopted practice, with fewer than one-third (29.55%) stating they used them every time (Figure 5).

### Factors associated with lifetime NSI (Bivariate analysis)

Bivariate analysis revealed two factors significantly associated with lifetime NSI: higher patient volume (treating >12 patients per day) was strongly associated (*p* < 0.001), and age showed a significant association (*p* = 0.004), with the highest NSI proportion in the youngest age group (20–29 years: 87.7%). No other demographic or professional characteristics reached statistical significance (Table 1).

**Table 1:** Association between Demographic, professional Factors and Lifetime Needlestick/Sharp Injury (NSI).

Variable	Category	Total (*n* = 423)	Yes (*n* = 373)	No (*n* = 50)	*p*-value
Gender	Male	139 (32.86)	124 (33.24)	15 (30.00)	0.728
	Female	284 (67.14)	249 (66.76)	35 (70.00)	
Age (years)	20–29	366 (86.52)	327 (87.67)	39 (78.00)	0.004^*^
	30–39	43 (10.17)	35 (9.38)	8 (16.00)	
	≥40	14 (3.31)	11 (2.95)	3 (6.00)	
Years in practice	<3 years	291 (68.79)	256 (68.63)	35 (70.00)	0.739
	3–10 years	112 (26.48)	99 (26.54)	13 (26.00)	
	>10 years	20 (4.73)	18 (4.83)	2 (4.00)	
Professional qualification	Student (5th year)	256 (60.52)	228 (61.13)	28 (56.00)	0.561
	Intern	98 (23.17)	87 (23.32)	11 (22.00)	
	GDP/Postgraduate/Specialist	69 (16.31)	58 (15.55)	11 (22.00)	
Practice setting	Academic	127 (30.02)	108 (28.95)	19 (38.00)	0.105
	Private practice	105 (24.82)	96 (25.74)	9 (18.00)	
	Public/Government	103 (24.35)	91 (24.40)	12 (24.00)	
	Mixed	88 (20.80)	78 (20.91)	10 (20.00)	
Work hours per day	≤8 h	244 (57.68)	211 (56.57)	33 (66.00)	0.101
	>8 h	179 (42.32)	162 (43.43)	17 (34.00)	
Patients per day	≤12 patients	288 (68.09)	244 (65.42)	44 (88.00)	<0.001^*^
	>12 patients	135 (31.91)	129 (34.58)	6 (12.00)	

P-values from chi-square test (Fisher's exact for “NSIs are unavoidable”).

* : statistical significance (p < 0.05).

Bivariate analysis of infection control practices, resource availability, and attitudes (Table 2) revealed that the belief that “NSIs are an unavoidable part of dental training” was significantly more common among those who had experienced an NSI (24.66% vs. 8.00%, *p* = 0.010). No self-reported infection control practices or resource availability variables showed a significant unadjusted association with NSI.

**Table 2:** Associations of infection control practices, resource availability, and attitudes with lifetime NSI.

Variable	NSI Yes (*n* = 373) *n* (%)	NSI No (*n* = 50) *n* (%)	*p*-value
Self-reported infection control practices			
Always wear gloves, mask, gown	318 (85.52)	45 (90.00)	0.513
Always use eye protection/face shield	146 (39.14)	26 (52.00)	0.120
Always use high-volume suction	187 (50.13)	29 (58.00)	0.156
Always one-hand recapping	190 (50.94)	25 (50.00)	0.154
Always use safety-engineered devices	107 (28.69)	18 (36.00)	0.332
Always dispose sharps immediately	294 (78.82)	41 (82.00)	0.534
Always avoid passing sharp instruments	290 (77.75)	41 (82.00)	0.294
Infection control resources at the training site			
Designated infection control unit/officer	236 (63.27)	30 (60.00)	0.511
Regular NSI prevention training	177 (47.45)	23 (46.00)	0.768
Adequate supply of PPE	257 (68.90)	33 (66.00)	0.686
Safety-engineered devices available	263 (70.51)	32 (64.00)	0.375
Perceptions of NSI prevention			
Agree: NSIs are unavoidable	92 (24.66)	4 (8.00)	0.010^*^
Agree: Reporting NSI is important	258 (69.17)	30 (60.00)	0.162
Agree: Anxious about BBV transmission	194 (52.10)	20 (40.00)	0.096^*^
Agree: Benefit from more hands-on training	216 (57.91)	28 (56.00)	0.762
Agree: Institution prioritizes safety	179 (47.99)	30 (60.00)	0.118

P-values from chi-square test (Fisher's exact for “NSIs are unavoidable”).

* : statistical significance (p < 0.05).

### Multivariable logistic regression analysis

Multivariable logistic regression was conducted to identify factors independently associated with lifetime NSI using the reduced predictor set (Table 3). After adjustment, treating more than 12 patients per day remained the only significant independent factor, with an adjusted odds ratio of 3.50 (95% CI 1.45–8.46, *p* = 0.005). No other variable—including age, years in practice, professional qualification, work hours, or the composite infection control practice score-reached statistical significance. The model explained 21.3% of the variance (Nagelkerke R^2^ = 0.213) and showed adequate fit (Hosmer-Lemeshow *p* = 0.642).

**Table 3:** Factors associated with lifetime NSI (multivariable logistic regression).

Variable	Variable	aOR	95% C		*p*-value
			Upper	Lower	
Age (years)	20–29	Ref.			
	30–39	0.71	0.27	1.84	0.480
	≥40	0.68	0.14	3.21	0.624
Years in practice	<3 years	Ref.			
	3–10 years	1.03	0.47	2.24	0.948
	>10 years	0.83	0.16	4.19	0.821
Professional qualification	Student (5th year)	Ref.			
	Intern	1.13	0.46	2.78	0.786
	GDP/Postgrad	0.86	0.35	2.09	0.738
Patients per day	≤12 patients	Ref.			
	>12 patients	3.50	1.45	8.46	0.005^*^
Work hours per day	≤8 h	Ref.			
	>8 h	1.57	0.80	3.10	0.188
Always use eye protection/face shield	No	Ref.			
	Yes	0.73	0.38	1.39	0.338
Always one-hand recapping	No	Ref.			
	Yes	1.32	0.69	2.52	0.402
Always use safety-engineered devices	No	Ref.			
	Yes	0.92	0.44	1.92	0.821

aOR = adjusted odds ratio; CI = confidence interval.

* = statistical significance, p < 0.05. (Model fit: Nagelkerke R^2^ = 0.213; Hosmer-Lemeshow goodness-of-fit test p = 0.642).

## Discussion

This cross-sectional study of 423 dental practitioners in Sana'a, Yemen, reveals an alarmingly high lifetime prevalence of needlestick and sharp injuries (88.2%), with nearly one-quarter of affected participants reporting four or more NSIs in the preceding year. Syringe needles were the most frequently implicated device, and injuries most commonly occurred during local anesthesia administration. Critically, while immediate wound care was common, only 11.3% of injured participants underwent post-exposure testing and only 9.4% received PEP. After multivariable adjustment, treating more than 12 patients per day was the sole independent predictor of NSI, increasing the odds more than threefold.

### Comparison with previous studies

The 88.2% lifetime prevalence observed in our study substantially exceeds most previously reported estimates. A 2022 systematic review and meta-analysis estimated global NSI prevalence among dental students at 44% (6). More recent studies continue to show wide variation: a 2025 Moroccan study reported 45.3% among dental students (10), while a 2023 Chinese study found 23.8% among dental interns (8).

The finding that syringe needles were the most common device (61.7%) and local anesthesia administration the most frequent procedure (39.1%) aligns closely with prior research identifying local anesthesia as a high-risk procedure (7, 16, 18). A five-year surveillance study at a Swiss dental school similarly found that syringe needles dominated occupational incidents (26). Notably, only 12.3% of injuries in our study occurred during recapping; the vast majority occurred during administration. This suggests that the actual process of injection—handling the syringe at chairside—may represent a more significant risk point than the traditionally emphasized hazard of recapping, a finding with important implications for training and device design.

A critical factor contributing to the overall high prevalence of needlestick injuries (NSIs) at 88.2% is the demographic makeup of the sample, consisting primarily of fifth-year dental students and interns (83.69% of respondents). Literature indicates that early-career practitioners face greater risks for NSIs due to ongoing clinical training and limited experience in managing complex procedures (10, 25). This overrepresentation of trainees inflates injury rates, which may not reflect those experienced practitioners. Additionally, the clinical settings surveyed, predominantly university dental hospitals and public facilities, complicate the injury rates further. These environments differ from private practices by involving high patient volumes, complex procedures, and varying faculty supervision levels. In resource-limited public and academic settings in Yemen, dental practitioners struggle with the availability of advanced infection control resources and safety-engineered devices. This combination of intense learning environments, high workloads, and scarce resources results in heightened occupational exposure risks.

### High patient volume as the principal risk factor

The independent association between treating >12 patients per day and NSI (aOR = 3.50) is a notable finding. This aligns with broader healthcare literature demonstrating that high-volume workloads and time pressure have a direct correlation with increased rates of percutaneous injuries (26–28). The mechanism whereby increased workload and time pressure lead to increased percutaneous injuries is likely the result of increased speed of procedure completion; performing procedures while fatigued; and reduced compliance with safe working practices due to time pressure (29, 30).

After adjusting for patient volume, none of the other variables examined were statistically significant predictors of percutaneous injuries, including years of experience, professional credentials, or self-reported adherence to safety procedures such as one-handed recapping or using safety-engineered devices. This may reflect that time pressure reduces adherence to safe practices, though observational studies are needed to confirm this hypothesis. Alternatively, it may indicate that self-reported compliance does not accurately reflect actual practice under time pressure. Observational studies have shown that as patient volumes increase, compliance with safety procedures is subsequently reduced (31). This assumption is consistent with the our data, because although 85.8% of participants reported “always” wearing personal protective equipment, and 79.2% of participants reported immediate disposal of sharp objects, the NSI rates remained very high, suggesting that self-reported adherence to safety procedures may be subject to substantial social desirability bias.

### Gaps in post-exposure management

While over half of the participants either cleaned the wound and/or used antiseptics, only 11.26% were tested for blood borne viruses (BBV), and only 9.38% were provided with post-exposure prophylactic medication (PEP). This is concerning in light of the fact that there was a known contamination of the device in 41.55% of cases, and that there was a lack of clarity regarding the contamination of the device in 38.61% of cases. Similar barriers to receiving PEP have been documented among health care workers worldwide. Barriers include: a lack of awareness of protocols; fear of side effects; a low perception of risk; or gaps associated with the ability of institutions to provide timely access to PEP (32, 33). Therefore, these findings suggest a need for strengthening post-exposure management training and ensuring that training sites have clear pathways for timely access to PEP.

### Attitudes toward NSI prevention

A notably interesting finding regarding attitudes was that 24.66% of those with NSI experience agreed that “NSIs are an unavoidable part of dental training,” compared with only 8.00% of those without NSI (*p* = 0.010). This implies that experiencing an injury could normalize the incident and create a sense of inevitability, undermining efforts to encourage prevention. A study conducted in Pakistan reported that students who had experienced NSIs were less likely to perceive them as preventable (34). Educational interventions have been shown to improve knowledge and decrease incidence of NSI; a prospective study conducted in Saudi Arabia demonstrated that receiving structured infection prevention training reported a reduction in NSIs from 17.2% to 4.3% (15).

### Implications for training and policy

This research has many real-world implications; First, limiting the number of patients assigned to trainees, especially in their early clinical training years, will help reduce injury rates. Second, healthcare facility administration should promote the adequate availability and proper usage of safety-engineered medical devices; in our study, only 29.6% of respondents reported always using safety-engineered devices. The World Health Organization (WHO) strongly advises healthcare providers to use safety-engineered syringes to minimize needles-stick injuries (35). Third, regular training sessions that incorporate opportunities for hands-on simulation of high-risk procedures and post-exposure protocols will be necessary (15). Finally, creating an environment of safety by promoting open reporting will be vital, as well as treating NSI as events that can be avoided rather than as accidents that are unavoidable (36).

### Study limitations

The present study has several limitations. First, its cross-sectional nature precludes drawing causal relationships. Second, the use of convenience sampling and voluntary participation introduces a strong potential for selection bias; it is highly plausible that Individuals with prior NSI experiences may have been more motivated to participate in an occupational safety survey, potentially inflating the observed prevalence. Although our sample was large (*n* = 423) and drawn from a variety of training sites, the findings may not be generalizable to rural settings or to practitioners in solo private practice. Third, the retrospective self-report of NSI occurrences may introduce recall bias, and social desirability bias may also affect reporting of safety measures. Recall bias may affect both the lifetime prevalence estimate and the details of the most recent injury. Specifically, participants may be more likely to recall and report injuries they have experienced, potentially overestimating the lifetime prevalence, while also underreporting events from earlier in their careers. Fourth, our sample was predominantly composed of dental students and interns, a group inherently at higher risk for NSIs due to limited clinical experience. The overrepresentation of this high-risk group limits the generalizability of our prevalence estimate to more experienced practitioners. In addition, the differences between university-based clinical training environments and private practice settings, including variations in supervision, workload, procedure complexity, patient volume, and availability of infection control resources, may influence injury risk as our mixed sample included practitioners from diverse settings. Fifth, under-reporting of NSI is well documented in the dental education literature (37, 38); the direction of bias in self-reported surveys is uncertain, and the true prevalence could be either higher or lower than the 88% we observed.

Additionally, our definition of “high patient load” (>12/day) was relatively simplistic; future studies should use more comprehensive workload measures that incorporate patient acuity, procedure complexity, and time-motion analysis. We did not measure fatigue, procedure complexity, clinical setting (e.g., emergency vs. routine), or supervision level, all of which may influence NSI risk. Finally, our statistical model explained only a modest share of the variance (Nagelkerke R^2^ = 0.213). This tells us that other factors we did not measure-like fatigue, distraction, specific clinical techniques, and the safety culture of the workplace—likely play a major role as well.

## Conclusion

Needlestick and sharp injuries are highly prevalent among dental practitioners in Yemen, with high patient volume as the key modifiable risk factor. The number of dental professionals who receive testing for bloodborne pathogens after an exposure and post-exposure prophylaxis (PEP) is also low. Interventions should be focused on managing workloads, using safety-engineered devices, and improving post-exposure protocols. The perception through educational programs about needlestick and sharp injuries (NSIs) as being “inevitable” should be shifted by promoting a preventive and timely reporting culture. Addressing these gaps is essential to protect practitioners and prevent bloodborne pathogen transmission.

## Data Availability

The original contributions presented in the study are included in the article/Supplementary Material, further inquiries can be directed to the correspondingauthors.

## References

[1] Mohamud RYH Mohamed NA Dogan A Hilowle FM Isse SA Hassan MY . Needlestick and sharps injuries among healthcare workers at a tertiary care hospital: a retrospective single-center study. Risk Manag Healthc Policy. (2023) 16:2281–9. doi: 10.2147/RMHP.S434315 37953810 PMC10637236

[2] Rashidov A Katib H Alem SK Al Harbi F Noor A Luna R. The epidemiology of needlestick and sharp injuries among healthcare workers in a secondary care hospital in saudi arabia: a retrospective study. Cureus. (2024) 16:e58880. doi: 10.7759/cureus.58880 38800323 PMC11116932

[3] Lee JH Cho J Kim YJ Im SH Jang ES Kim JW . Occupational blood exposures in health care workers: incidence, characteristics, and transmission of bloodborne pathogens in South Korea. BMC Public Health. (2017) 17:827. doi: 10.1186/s12889-017-4844-0 29047340 PMC5648449

[4] Marji T Syed MA. Primary care dental professionals' experiences of sharp injuries in Qatar: a cross-sectional study. Front Oral Health. (2022) 3:1014004. doi: 10.3389/froh.2022.1014004 36532093 PMC9755168

[5] Bouya S Balouchi A Rafiemanesh H Amirshahi M Dastres M Moghadam MP . Global prevalence and device related causes of needle stick injuries among health care workers: a systematic review and meta-analysis. Ann Glob Health. (2020) 86:35. doi: 10.5334/aogh.2698 32346521 PMC7181946

[6] Huang J Li N Xu H Liu Y An N Cai Z. Global prevalence, risk factors, and reporting practice of needlestick and sharps injuries among dental students: a systematic review and meta-analysis. J Hosp Infect. (2022) 129:89–101. doi: 10.1016/j.jhin.2022.06.015 35781020

[7] Zeng J Li E Xu Y Lin Y Xiao Y Yu X. Prevalence of needlestick injuries in dental assistants: a systematic review and meta-analysis. J Glob Health. (2025) 15:04030. doi: 10.7189/jogh.15.04030 39950562 PMC11826961

[8] Huang J Gan Y Xu H Li N An N Cai Z. Prevalence and characteristics of needlestick injuries among dental interns during their first-year clinical training: an observational study. BMC Oral Health. (2023) 23:194. doi: 10.1186/s12903-023-02892-5 37009865 PMC10067515

[9] Shaghaghian S Golkari A Pardis S Rezayi A. Occupational exposure of shiraz dental students to patients' blood and body fluid. J Dent. (2015) 16:206–13. PMC4554314 26331151

[10] Amminou L Soualem H Boukssim S Chbicheb S. Blood exposure accidents and associated risk factors among dental students in Rabat, Morocco: a cross-sectional study. Infect Prev Pract. (2025) 8:100498. doi: 10.1016/j.infpip.2025.100498 41488818 PMC12756618

[11] Kofman AD Struble KA Heneine W Gayle B de Perio MA Okasako-Schmucker DL . 2025 US Public Health Service Guidelines for the Management of Occupational Exposures to Human Immunodeficiency Virus and Recommendations for Post-exposure Prophylaxis in Healthcare Settings. Infect Control Hosp Epidemiol. (2025) 46:863–73. doi: 10.1017/ice.2025.10254 41569270 PMC12616222

[12] Zhang M Chen Y Zhang S Fan X. Sharps injuries in a dental specialty hospital: retrospective analysis of occupational risks, 2020-2024. BMC Oral Health. (2025) 25:1618. doi: 10.1186/s12903-025-07020-z 41088086 PMC12523194

[13] Azab E Afifi IK. Awareness and reporting of sharps injuries: a study involving dental students, trainees, and assistants in a dental teaching hospital in Saudi Arabia. Cureus. (2024) 16:e52843. doi: 10.7759/cureus.52843 38268990 PMC10807201

[14] Elisa N Ssenyonga L Iramiot JS Nuwasiima D Nekaka R. Sharp/Needlestick injuries among clinical students at a tertiary hospital in Eastern Uganda. medRxiv (Preprint). 2023 Feb 2:2023.02.01.23285330. Update in: J Infect Prev. *Nov*. (2025) 5:17571774251394873. 10.1177/17571774251394873 PMC12588969 41210190

[15] Alshammari AF Madfa AA Alenezi YE Alshammari HM Alazaima DM Alotaibi AS . The potential benefit of an educational intervention on needle stick injury prevention among dental students at the university of Ha'il, Saudi Arabia: a prospective study. BMC Med Educ. (2025) 25:1026. doi: 10.1186/s12909-025-07581-1 40634919 PMC12243357

[16] AlDakhil L Yenugadhati N Al-Seraihi O Al-Zoughool M. Prevalence and associated factors for needlestick and sharp injuries (NSIs) among dental assistants in Jeddah, Saudi Arabia. Environ Health Prev Med. (2019) 24:60. doi: 10.1186/s12199-019-0815-7 31601166 PMC6788026

[17] Halboub ES Al-Maweri SA Al-Jamaei AA Tarakji B Al-Soneidar WA. Knowledge, Attitudes, and Practice of Infection Control among Dental Students at Sana'a University, Yemen. J Int Oral Health. (2015) 7:15–9. 26028896 PMC4441229

[18] Ravi A Shetty PK Singh P Wakode D Modica SF Kodaganallur Pitchumani P . Needlestick injuries in dentistry: time to revisit. J Am Dent Assoc. (2023) 154:783–94. doi: 10.1016/j.adaj.2023.06.004 37530693

[19] Parums DV. Editorial: The 2024 revision of the declaration of helsinki and its continued role as a code of ethics to guide medical research. Med Sci Monit. (2024) 30:e947428. doi: 10.12659/MSM.947428 39616449 PMC11619173

[20] Daniel WW Cross CL. Biostatistics: A Foundation for Analysis in the Health Sciences. 11th ed. Hoboken, NJ: Wiley. (2019).

[21] Pavithran VK Murali R Krishna M Shamala A Yalamalli M Kumar AV. Knowledge, attitude, and practice of needle stick and sharps injuries among dental professionals of Bangalore, India. J Int Soc Prev Community Dent. (2015) 5:406-12. doi: 10.4103/2231-0762.165932 26539394 PMC4606606

[22] Trayner KMA Hopps L Nguyen M Christie M Bagg J Roy K. Cross-sectional survey of a sample of UK primary care dental professionals' experiences of sharps injuries and perception of access to occupational health support. Br Dent J. (2018) 225:1031–1036. doi: 10.1038/sj.bdj.2018.1031 30499564

[23] Zara B Shuja E Um Min Allah N Pervez M Siddiquie O Siddique S. Needlestick injuries among dental professionals in dental colleges of Rawalpindi, Pakistan. J Bahria Univ Med Dent Coll. (2020) 10:181–7. doi: 10.51985/JBUMDC2019003

[24] Hakimi-Meibodi MR Rezaei F Hatami M Owlia F. Evaluating the knowledge and practice of Yazd general dentists toward needle stick injuries (NSI) in 2022. J Health Field. (2023) 11.

[25] Dukka H Byrd P Qian C Baughman G Butt S Rai SN. Occupational percutaneous injuries and exposures in a dental teaching environment: a 10-year report. J Dent Educ. (2021) 85:1729–38. doi: 10.1002/jdd.12731 34180052

[26] Kulik EM Bornstein MM. Needlestick injuries and related occupational accidents with sharp objects in a dental school. Swiss Dent J. (2025) 135:188-201. doi: 10.61872/sdj-2025-01-07 40103398

[27] Hassanipour S Sepandi M Tavakkol R Jabbari M Rabiei H Malakoutikhah M . Epidemiology and risk factors of needlestick injuries among healthcare workers in Iran: a systematic reviews and meta-analysis. Environ Health Prev Med. (2021) 26:43. doi: 10.1186/s12199-021-00965-x 33794759 PMC8015057

[28] Alfulayw KH Al-Otaibi ST Alqahtani HA. Factors associated with needlestick injuries among healthcare workers: implications for prevention. BMC Health Serv Res. (2021) 21:1074. doi: 10.1186/s12913-021-07110-y 34627244 PMC8502299

[29] Joukar F Mansour-Ghanaei F Naghipour M Asgharnezhad M. Needlestick Injuries among Healthcare Workers: Why They Do Not Report their Incidence? Iran J Nurs Midwifery Res. (2018) 23:382-387 doi: 10.4103/ijnmr.IJNMR_74_17 30186344 PMC6111658

[30] Dulon M Stranzinger J Wendeler D Nienhaus A. Causes of needlestick and sharps injuries when using devices with and without safety features. Int J Environ Res Public Health. (2020) 17:8721. doi: 10.3390/ijerph17238721 33255337 PMC7727709

[31] Charles C Mkonongo L Masanja D Maruba D Mwita PF Nkondo B . Healthcare workers' perspectives on factors influencing compliance with infection prevention and control practices at Katavi Regional Referral Hospital, Tanzania. Hygiene. (2026) 6:17. doi: 10.3390/hygiene6010017 PMC13258126 42275309

[32] Bun RS Aït Bouziad K Daouda OS Miliani K Eworo A Espinasse F . Identifying individual and organizational predictors of accidental exposure to blood (AEB) among hospital healthcare workers: A longitudinal study. Infect Control Hosp Epidemiol. (2024) 45:491–500. doi: 10.1017/ice.2023.248 38086622 PMC11007361

[33] Auerbach JD Malone S Forsyth AD. Occupational post-exposure prophylaxis among healthcare workers: a scoping review of factors affecting optimal utilization. J Int AIDS Soc. (2024) 27:e26341. doi: 10.1002/jia2.26341 39155429 PMC11330849

[34] Arif F Safi S Ullah H Waqar H Qanita M. Evaluating dental students' knowledge, attitude and practice of needle stick injuries. J Rehman Coll Dent. (2025) 6:82–8. doi: 10.52442/jrcd.v6i03.136

[35] WHO Guideline on the Use of Safety-Engineered Syringes for Intramuscular, Intradermal and Subcutaneous Injections in Health-Care Settings. Geneva: World Health Organization (2015). 26203487

[36] Zachar JJ Reher P. Percutaneous exposure injuries amongst dental staff and students at a university dental clinic in Australia: a 6-year retrospective study. Eur J Dent Educ. (2022) 26:288–95. doi: 10.1111/eje.12701 34117686

[37] Voide C Darling KE Kenfak-Foguena A Erard V Cavassini M Lazor-Blanchet C. Underreporting of needlestick and sharps injuries among healthcare workers in a Swiss University Hospital. Swiss Med Wkly. (2012) 142:w13523. doi: 10.4414/smw.2012.13523 22328010

[38] Pervaiz M Gilbert R Ali N. The prevalence and underreporting of needlestick injuries among dental healthcare workers in Pakistan: a systematic review. Int J Dent. (2018) 2018:9609038. doi: 10.1155/2018/9609038 29623091 PMC5829343

