# Supplementary material for: Needlestick and sharps Injuries among dental practitioners: a cross-sectional study of prevalence, risk factors, and postexposure management
Source: Front Public Health. 2026 Jul 13;14:1876433. doi: 10.3389/fpubh.2026.1876433 (PMC13402525; doi:10.3389/fpubh.2026.1876433)
Supplement: Supplementary file 1 [file Supplementary_file_1.docx]

**Title: Title: Needlestick and Sharps Injuries among Dental Practitioners: A Cross-sectional Study of Prevalence, Risk Factors, and Post-exposure Management**

*Dear Participant*

This questionnaire designed to assess the occurrence and associated factors of needle stick and sharp injuries (NSIs) among dental practitioners.

Your participation is voluntary, anonymous, and confidential.

The survey takes ~5–8 minutes. Participation is voluntary; responses are anonymous and will be used for research only. Please answer honestly.

**Section1:Demographic and Academic Information**

*(****Instructions****: Please provide your background details by selecting the option that best describes you)*.

**1. Gender:**

☐ Male ☐ Female

**2. Age (in years)?**

□ 20–29 □ 30–39 □ ≥40

**3. Number of years in practice?** □ <3 years □ 3–10 years □ > 10 years

**4. What is your highest educational qualification?**

□ 5 th year dental student □ Intern □ GDP □ Postgraduate □ Specialist

**5. Type of Practice:**

☐ Public/Government practice ☐ Private practice ☐ Academic ☐ Mixed

**6. On average, how many hours do youwor kper day?**

□ ≤ 8 hours □ > 8hours

**7. On average, how many patients do you attend per day?**

□ ≤12 patients □ > 12 patients

**Section 2 :History of Needle stick and Sharp Injuries (NSIs)**

*(****Instructions:*** *This section asks about any past injuries involving needles or sharp instruments, Please select the most appropriate option for each question)*

**8. Have you ever experienced a needle stick or sharp injury during dental training?**

☐Yes. ☐ No *(if No, skip to Section 3)*

**9. Number of NSIs experienced in the past 12 months:**

☐ None. ☐ 1 ☐ 2–3 ☐ ≥4

**10. What device was involved in your most recent NSI? *(select only one)*?**

☐ Syringe needle. ☐ Dental bur/ instrument.

☐ Scalpel / surgical blade ☐ Endodontic file.

☐ Suture needle. ☐ Other

**11. What procedure were you performing at the time of your most recent NSI? *(select one)*:**

☐ Local anesthesia administration. ☐ Surgical extraction

☐ Suturing. ☐ Recapping needle

☐ Scaling and root planing. ☐ Other procedures

**12. Was the device contaminated with blood or saliva at the time of your most recent NSI?**

☐ Yes. ☐ No. ☐ Unsure

**13. Which part of your body was injured in your most recent NSI?**

□ Finger (s) □ Hand. □ Arm □ Other

**14. What immediate post‑exposure action(s) did you take after your most recent NSI? *(select all that apply)*:**

☐ Washed the wound with soap and water

☐ Applied an antiseptic(e.g., alcohol, iodine)

☐ Received Post-Exposure Prophylaxis (PEP)

☐ Got screened for HBV/ HCV/HIV

☐ No action taken

**Section 3: Compliance with Infection Control Practices**

(***Instructions***: *Please indicate how often you perform each of the following practices during routine patient care).*

**15. Wear gloves, mask, and gown during patient care?**

☐ Always

☐ Some times

☐ Never

**16. Use eye protection or face shield?**

☐ Always

☐ Some times

☐ Never

**17. Use high-volume suction to reduce aerosol?**

☐ Always

☐ Some times

☐ Never

**18. Recap needles using the one-hand technique?**

☐ Always

☐ Some times

☐ Never

**19. Use safety-engineered devices (e.g., safety syringes, retractable needles)?**

☐ Always

☐ Some times

☐ Never

**20. Dispose of sharps immediately after use?**

☐ Always

☐ Some times

☐ Never

**21. Avoid passing sharp instruments by hand?**

☐ Always

☐ Some times

☐ Never

**Section 4 :Institutional and Environmental Factors**

***(Instructions****:* ***Please respond based on the availability of safety resources at your clinical training site)*.**

**Does your clinical training site have?:**

**22. Designated infection control unit/officer:** ☐ Yes ☐ No

**23. Regular NSI prevention training:**  ☐ Yes ☐ No

**24. Adequate supply of personal protective equipment (PPE):** ☐ Yes ☐ No

**25. Safety-engineered devices available:**  ☐ Yes ☐ No

**Section 5:Attitudes and Perceptions**

*(****Instructions:*** *Please Share your personal views by indicating your level of agreement with each statement).*

**26. NSIs are an unavoidable part of dental training.**

☐ Strongly Disagree

☐ Disagree

☐ Neutral

☐ Agree

☐ Strongly Agree

**27. Reporting an NSI is important even if the device was uncontaminated.**

☐ Strongly Disagree

☐ Disagree

☐ Neutral

☐ Agree

☐ Strongly Agree

**28. I feel anxious about contracting HBV/HCV/HIV from an NSI.**

☐ Strongly Disagree

☐ Disagree

☐ Neutral

☐ Agree

☐ Strongly Agree

**29. I would bene fit from more hands-ontraining in NSI prevention.**

☐ Strongly Disagree

☐ Disagree

☐ Neutral

☐ Agree

☐ Strongly Agree

**30. My institution prioritizes practitioner safety adequately.**

☐ Strongly Disagree

☐ Disagree

☐ Neutral

☐ Agree

☐ Strongly Agree

*Thank you for your participation*
